# Measured depth of subcutaneous tissue on posterolateral arm of omalizumab patients

**DOI:** 10.1186/1710-1492-8-S1-A5

**Published:** 2012-11-02

**Authors:** Ryan Potts, Laura Kim, Clark Eeuwes, Arunmozhi Dominic, Immaculate Nevis, Harold L Kim

**Affiliations:** 1 University of Waterloo, Waterloo, Ontario, Canada; 2 McGill University, Montréal, Québec, Canada; 3 University of Western Ontario, London, Ontario, Canada; 4 McMaster University, Hamilton, Ontario, Canada

## Background

Omalizumab is a humanized antibody utilized for patients with moderate/severe allergic asthma. There is an estimated risk of anaphylaxis to omalizumab in 0.1% of patients. Omalizumab should be received in the subcutaneous tissue of the mid-posterolateral upper arm. There may be an increased risk of anaphylaxis if injections are received intramuscularly (IM). In our clinic, omalizumab is given with *BD Eclipse™ Needle*, which is routinely provided with the drug and has needle length 16mm. If a patient has a skin to muscle depth (STMD) less than 16mm, there is a risk of omalizumab being injected IM.

## Methods

We reviewed charts in an allergy clinic where an ultrasound of the left posterolateral arm was completed to measure STMD. Patients were divided into two groups based on their STMD (>16 mm and ≤16 mm) and baseline characteristics were compared. We conducted multivariable linear regression with age, sex, BMI and race. The percentages of patients with STMD greater than 4 mm, 6 mm, 8 mm, 10 mm, and 12 mm were determined.

## Results

Ultrasounds were completed on 40 patients receiving omalizumab. Three (7.5%) patients examined had >16 mm of STMD. Baseline characteristics were consistent between the groups except for BMI (*p* < 0.05). Sex and BMI correlated with STMD based on the linear regression analysis. Also, 35 (87.5%) patients had >4 mm STMD.

## Conclusion

With provided omalizumab needles, the risk of anaphylaxis may be increased as the injections may be given IM. By reducing the needle length to 4 mm, the risk will likely be reduced.

